# Analyzing the Mechanical Properties of Free-Standing PACA Thin Films Using Microindentation Technique

**DOI:** 10.3390/polym14224863

**Published:** 2022-11-11

**Authors:** Osamah Altabal, Christian Wischke

**Affiliations:** Institute of Active Polymers, Helmholtz-Zentrum Hereon, Kantstr. 55, 14513 Teltow, Germany

**Keywords:** poly(alkyl cyanoacrylate), free-standing thin films, polymer mechanics, microindentation

## Abstract

Assessing the mechanical properties of materials is of fundamental relevance for their rational usage, but can be challenging with standard tensile testing for highly brittle polymers used, e.g., as coatings. Here, a procedure for the mechanical analysis of free-standing poly(alkyl cyanoacrylate) (PACA) films using microindentation has been explored. Rigid and transparent films from PACA with various side chain compositions were formed on top of square polymer frames by in situ polymerization. Under microscopic control, the free-standing films were analyzed using a microelectromechanical sensing system. By this procedure, decreasing Young’s moduli *E* for increasing PACA side chain length and flexibility were determined with strain at break *ε*_B_ between 0.36% for poly(ethyl cyanoacrylate) and 4.6% for poly(methoxyethyl cyanoacrylate). Based on this successful application, the applied methodology may be relevant for characterizing various coating materials, which are otherwise hard to form as thin free-standing films, and using the data, e.g., in computationally assisted design and evaluation of hybrid material devices.

## 1. Introduction

Brittle materials experiencing fatigue after only a few loading cycles and/or low relative deformations are typically not considered to be functional materials. In contrast to such negative reception of brittle materials for load-bearing purposes, brittleness can be advantageous for the creation of functional systems from coated devices, e.g., the creation of specific surface patterns [1] or nanowires [2], as well as the fabrication of nanofluidics molds [3]. A key requirement for a rational selection of coating materials is the understanding of their mechanical properties.

Poly(alkyl cyanoacrylate)s (PACAs) are examples of brittle materials, where the variation of alkyl side chains allows alteration of glass transition temperatures [4] and mechanical properties. Alkyl cyanoacrylate (ACA) monomers are characterized by an excellent spreadability, a prompt anionic polymerization in the presence of traces of water at ambient conditions, and hydrophobicity with low aqueous swellability of the formed polymerized products. ACA monomers are widely used in technical and medical applications, e.g., as glues for wire fixation or retention of nuts in vibrating devices, for superhydrophobic water-repellent surfaces [5], as bio-adhesives in wound closure interventions [6], repair of corneal perforations [7], and for hemostasis in dentistry [8]. PACAs have been further explored as nano-carriers for drug delivery applications with some tissue specificity [9,10] and as shell materials covering gas phase droplets (microbubbles) for ultrasound-triggered bursts [11]. Understanding structure–property relationships is key when aiming to tailor PACA-based materials to the specific needs of these or other applications. Beside processing techniques to control, e.g., material thickness, chemical approaches have been used to tailor material properties and increase its flexibility, e.g., by copolymerization of different ACA monomers, by PACA synthesis as semi-interpenetrating networks with oligo(ethylene glycol) [12] or the co-polymerization of ACA with flexible bifunctional linkers [13]. Again, given that brittleness may positively (e.g., ultrasound sensitive release) or negatively (integrity of self-cleaning superhydrophobic coatings) affect material functionality, an assessment of mechanical properties of thin-layer PACA-based materials would be vital to predict its later performance.

Assessing the mechanical properties of brittle materials such as PACAs, and thus concluding on structure–property relationships, can be challenging. Coating materials are often too thin and not strong enough to function alone in mechanical testing. Further challenges arise when these materials are hard to obtain as free-standing films and/or are brittle, which means that they may break during clamping in conventional tensile testing instruments. This has led to analytical strategies of combining the material of interest with ductile support materials [14], and investigating the composites using micro-/nanoindentation [15,16] or in wrinkling/cracking experiments [17]. Thin or ultrathin films of hydrophobic polymers can also be investigated in unilateral stretching when floating on water as a support material [18,19]. For coatings, tribological properties and adhesion of the different layers can also be studied in scratching tests [20]. So far, PACA mechanics have often been evaluated by applying ACA monomer as a glue between two sides of specimens, which are subjected to a load, e.g., by shearing or peeling [21,22].

Given the interest in PACAs as thin-layer materials for coatings and capsules, we here propose a methodology to form free-standing PACA films and evaluate their mechanical properties in the elastic regime as well as their ultimate strength. This analytical strategy has been based on the in situ polymerization of PACA on top of square-shaped polymer skeletons initially filled with a dissolvable matrix, followed by indentation of the free-standing polymer films with a microelectromechanical system (MEMS). A simple geometrical model allowed us to correlate the mechanical displacement and the detected stress to calculate the Youngs’s modulus *E* of thin-layer PACAs. In this way, structure–property relationships of the investigated series of PACAs could be demonstrated and the suitability of the testing concept could be verified to prepare and analyze thin free-standing films.

## 2. Materials and Methods

### 2.1. Materials

The used materials were negative photoresist (SU-8 2150) and developer (mr-Dev 600) from Micro resist technology GmbH, Berlin, Germany, polydimethyl siloxane (PDMS) (Sylgard 184) from Dow Corning GmbH, Wiesbaden, Germany, Hexane (CHROMASOLV, for HPLC, >97.0% (GC) from Sigma-Aldrich, Taufkirchen, Germany, dopamine hydrochloride from Alfa Aesar, Kandel, Germany, poly(ethylene glycol) (PEG) with an average molecular weight of $\bar{M_{w}}$ 1000 (PEG1000) from Acros Organics, part of Thermo Fisher Scientific, Hennigsdorf, Germany, methyl cyanoacrylate (Loctite 493), ethyl cyanoacrylate (Sicomet 40) and methoxyethyl cyanoacrylate (Loctite 460) from Henkel AG & Co, Düsseldorf, Germany. Samples of *n*-butyl cyanoacrylate (Cyberbond 7000) and ethoxyethyl cyanoacrylate (Cyberbond 5005) were kindly donated by Cyberbond Europe GmbH, Wunstorf, Germany. Table 1 shows the chemical structure of used CA monomers.

### 2.2. Fabrication of Polymer Substrates as Sample Holders

Square-shaped polymeric structures were fabricated by the photolithography-replication method. A photomask with a square frame pattern was designed using Autodesk (AutoCAD) software and printed at high resolution (128 K dpi; JD Photo Data company, Hitchin, UK). The frame templates were fabricated in a glass Petri dish (soda-lime glass, 60 × 15 mm, Duran group, Wertheim, Germany) by casting SU-8 2150 photoresist equal to 3 mm thickness followed by heating in the dark to 100 °C for 24 h (ramping rate ≈ 1.5 °C/min, hotplate HP 30 A, BlackHole Lab, Paris, France). The photomask and a UV filter (cut-on > 365 ± 7 nm, Laser Components GmbH, Olching, Germany) were placed on the photoresist, which was exposed to UV-light irradiation (6 irradiation cycles of 45 s with 5 min rest; mercury arc lamp 365 nm, intensity 114 mW/cm^2^; solar simulator system, Abet technology, Milford, CT, USA). After exposure, the Petri dish was heated at 95 °C for 30 min. Subsequently, the non-crosslinked photoresist was removed using developer solution (mr-Dev 600; 3 min exposure, total of 10 repeats) and the structure was rinsed with isopropanol and heated at 95 °C for 1 h in order to harden the photoresist.

Using the frame templates, casting molds (negatives) were prepared. PDMS base and curing agent (Sylgard 184) were mixed (10:1 w/w), poured onto the patterned frame templates in glass Petri dishes and degassed under vacuum for 10 min. The mixture was cured in an oven at 65 °C for 2 h and thereafter carefully peeled from the pattern, resulting in PDMS casting molds imprinted with a negative of the desired design.

**Figure 1:**
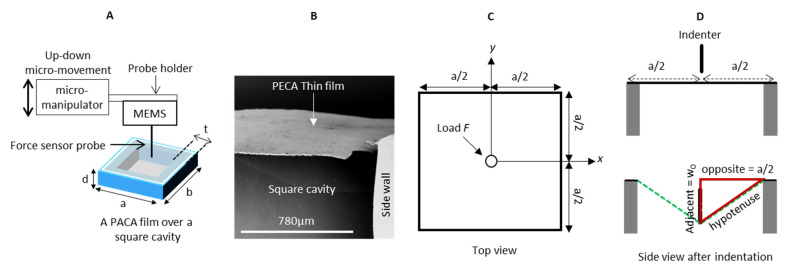
Test principle. (**A**) A schematic illustration of the microindentation setup. A square-shaped polymer frame (3 mm by 3 mm) covered with the PACA membrane is subjected to indentation with a MEMS device. (**B**) SEM image of an experimentally obtained PECA thin film over the square skele-ton. (**C**,**D**) Geometry model of the thin film from top and side view. (**D**) Upper panel: before indentation, lower panel: after indentation.

In a third step, square-shaped samples holders (positives) were prepared by melting (80 °C) oligo(*ε*-caprolactone)-diols (oCL) (CAPA 2803, Perstorp UK Ltd., Warrington, UK; number average molecular weight $\bar{M_{w}}$ 8 kDa) in the PDMS template, followed by removal of excess material, solidification at ambient conditions, and collection of the oCL sample holders (Figure 1A) from the PDMS mold using tweezers. Polydopamine as an adhesive material was deposited on the sample holder by immersion in 2 mL dopamine hydrochloride (2 mg/mL in tris(hydroxymethyl)aminomethane (TRIS) buffer 10 mM, pH = 8.5) under shaking at 150 rpm (IKA KS 260 Basic, IKA^®^ Werke GmbH & Co.KG, Staufen, Germany) for 24 h at room temperature (≈25 °C). Then, the square sample holders were rinsed with water and dried with a mild air stream (oil-free compressed air).

### 2.3. In Situ Formation of PACAs Films

Thin PACA films were prepared on top of the sample holders after their intermediate filling with PEG as a water soluble polymer. About 22 μL of molten PEG1000 (at 40 °C) were inserted into the square frames and solidified by cooling to ambient temperature (≈25 °C). On top of each filled frame, 1.5 μL of CA-monomer solution (1% *v/v* in hexane) was added and hexane was evaporated. After 16 repeats of CA deposition, the samples were placed under a fume hood for 24 h, where CA polymerized. Afterwards, each sample was carefully immersed in 10 mL water in a glass Petri dish (soda-lime glass, 60 × 15 mm, Duran group, Wertheim, Germany) under shaking for 1 h (50 rpm, IKA KS 260 Basic, IKA^®^ Werke GmbH & Co.KG, Staufen, Germany) to dissolve and remove the PEG filling. Samples with free-standing PACA films on top of frame sample holders were collected and dried under the fume hood.

### 2.4. Mechanical Analysis by Microindentation

The setup of the micro-indentation instrument consisted of an inverted microscope (Leica DMI6000B, Leica Microsystems, Wetzlar, Gemany), a MEMS micro-force sensor probe (FT-S10000-TP with a spherical glass tip of 25 μm, FemtoTools, Buchs, Switzerland), which was linked to a micromanipulator with a stepper motor controller (micromanipulator SM 3.25 with Tango 3 desktop controller, Märzhäuser Wetzlar GmbH, Wetzlar, Germany). The PACA membrane sample was placed on the microscope stage and the force sensor tip was centered over the PACA membrane. The contact point between the membrane and the tip was determined from the jump in the curve of the recorded force signal. For loading–unloading in the elastic regime, an indentation velocity of 5 μm/s was selected, while the velocity was 20 μm/s for the experiments with indentation up to PACA breakage.

### 2.5. Characterization of the Thickness of PACA Films

After each indentation experiment, samples were cut, vacuumed for 24 h and sputter-coated with gold (thickness of 5 nm; 5 × 10^−2^ mbar, 10–20 mA, 90 s; sputter coater SC7640, Quorum Technologies Ltd., Lewes, UK). The thickness of the different PACA films was examined by scanning electron microscopy (Phenom G2 pro, Phenom-World B.V, Eindhoven, The Netherlands).

### 2.6. Characterization of the Thermal Properties of PACA Films

PACA films for thermal analysis were prepared by casting PEG1000 at 40 °C in a Petri dish (diameter 3.5 cm), followed by 16 repeats of deposition of 160 µL of CA monomer solution (1% *v/v* in hexane) on the solidified PEG (compare Section 2.3). After immersion in water, PACA films were detached and floated, allowing collection in a tube, three repeats of washing with water and centrifugation (8500 rpm, Biofuge Stratos, Heraeus Instruments, Hanau, Germany), and finally freeze drying (Alpha 1-2LD plus, Christ, Osterode, Germany).

PACA films (≈5 mg) were analyzed using DSC (204 F1 Phoenix, Netzsch, Selb, Germany) in a temperature range of −100 to +150 °C for determining the glass transition temperature. Heating and cooling rates were 10 K·min^−1^ for PMCA, PECA and PBCA and 20 K·min^−1^ for PMECA and PEECA (as no signals were detectable at 10 K·min^−1^). The *T_g_* was determined at the inflection point of the thermograms from the second heating run.

## 3. Results and Discussion

### 3.1. Microindentation Setup and Model

In order to analyze the mechanical properties of free-standing PACA films, a methodology was proposed and evaluated. The principle bases on the in situ polymerization of PACA on specifically prepared small dimension frames (sample holders), which are investigated by an instrumentation comprising MEMS micro-force-sensing probes connected to a micromanipulator under an inverted microscope, as schematically illustrated in Figure 1A.

A square-like polymeric frame was selected as an exemplary sample holder that allows for both experimental handling and data analysis. The frames were obtained using photolithography and were microscopically analyzed to confirm their dimensions: the length *a* equaled the width *b*, each being 3 ± 0.1 mm. The frame depth *d* was 2.55 ± 0.03 mm and the wall thickness *t* was 0.3 ± 0.02 mm (*n* = 6). 

PACA films were formed on top of the sample holders via in situ polymerization of CA (Figure 1B), in this way defining the shape of the free-standing film as a square. The films adhered well on the walls of the sample holder during different preparation steps, including immersion in water, drying, etc. Given their square shape, a deformation model for membranes clamped at the edges could be adapted [23]. Upon indentation of the PACA film in the middle of the frame (Figure 1C), the centered displacement (*w*_0_) was linked to the applied force (*F*), the side length of the square (*a*) and the variables *ϑ* and *k* as expressed by Equation (1):

(1) $$w_{0}=\vartheta \frac{Fa^{2}}{k}$$

where *ϑ* is the numerical factor that depends on the geometry and is *ϑ* = 0.005602 for square-shaped films. k is the bending rigidity defined according to Equation (2):

(2) $$k=\frac{Eh^{3}}{12\left(1-\nu \right)}$$

where h is the thickness of the PACA membrane and ν is the Poisson’s ratio of the film material, assuming ν=0.4 for CA-based materials [24]. Substituting k in Equation (1) gives Equation (3). Accordingly, E can be calculated as given in Equation (4):

(3) $$w_{0}=\frac{\vartheta \;F\;a^{2}\;12\;\left(1-\nu \right)}{E\;h^{3}}$$

(4) $$E=\frac{\vartheta \;F\;a^{2}\;12\;\left(1-\nu \right)}{h^{3}\;w^{0}}$$

In order to quantify the ultimate strength, the centered displacement of the film was converted to a strain value by applying Pythagoras’s theorem of a triangle $\;(hypotenuse^{2}=adjacent^{2}+opposite^{2}$). During the indentation at the center of the laterally fixed thin film, it stretched to form a triangular shape at a cross-sectional view (red triangle in Figure 1D). The opposite and the adjacent of the triangle are known values, which refer to the half-length of the film (*a*/2) and to *w*_0_, respectively. The hypotenuse of the triangle correlates to the stretched length *a*/2 of the coating film and thus the strain can be calculated according to Equation (5):

(5) $$Strain\%=\frac{\left(2\;\cdot \;hypotenuse\right)-a}{a}\;\cdot \;100\;\;=\frac{\left(2\cdot \sqrt{{\left(0.5\;a\right)}^{2}+{\left(w_{0}\right)}^{2}\;}\right)-a}{a}\;\cdot \;100$$

### 3.2. Elastic Deformation Characteristics of PACA Depending on Polymer Side Chains

Small-amplitude mechanical deformations are typically accepted by materials, including brittle materials, as long as they are in the elastic deformation range. Simulating such small deformations in the proposed setup should allow for determining the elastic properties, especially the Young’s modulus *E* as an important material characteristic. Given the effect of PACA structure, particularly the length and type of alkyl or alkoxy side chains, on polymer-free volumes, thermal properties and brittleness [25,26,27], it was interesting to evaluate if the proposed setup would be suitable for detecting differences in the *E* moduli of free-standing films.

**Figure 2:**
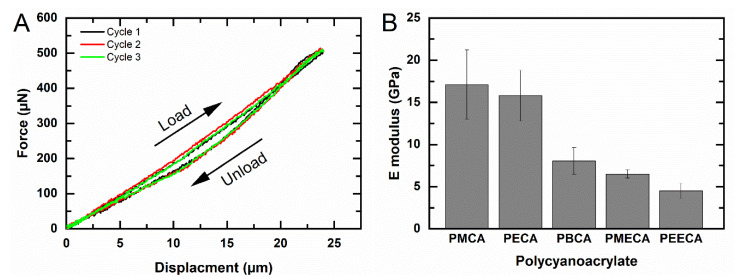
(**A**) Load–unload curve by indentation of PECA membrane over the square polymer skeleton (typical experimental error of the force sensor probe is 0.5 mN). (**B**) Elastic modulus *E* of free-standing PACA films with different side chains. Data represented as mean ± SD (*n* = 3). Data quantified by Equation (4).

In the first step, it should be tested if deposited films can be subjected to several cycles of mechanical loading without breakage or detachment. This is a prerequisite for applying Equation (4), which is only valid in the elastic deformation range under a small strain when the thin film remains fixed at edges of the polymer frame. The obtained load–unload curves from microindentation of PECA membrane (indented up to 25 µm in 3 cycles) are shown in Figure 2A. The loading curves were almost linear, while the unloading showed a minor hysteresis. This pattern indicated a viscoelastic behavior of PECA at this indentation range, where the small area within the hysteresis loop may be attributed to energy dissipation caused by internal friction [28]. By releasing the indentation load, the measured force returned to zero. Additional loading cycles showed congruent curves without shifts. This indicated a full recovery of the sample at the selected displacement range to its initial state and additionally confirmed that no delamination occurred during the indentation experiments, i.e., the sample remained fixed at the polymer frame.

In the second step, the *E* moduli should be assessed. The mechanical properties of polymers can strongly depend on the environmental temperature and may significantly shift in the temperature range of thermal transitions, such as the glass transition temperature ($T_{g}$). In order to exclude the thermal effect, all indentation experiments were performed at ambient temperature (≈25 °C), which is below the $T_{g}$ of all examined PACA films (Table 2). Thus, all analyzed PACA films were rigid and transparent. The thickness of each PACA film was characterized by SEM as summarized in Table 2. The E modulus was determined from the linear loading curve of the respective samples, clearly confirming that an increasing length of the alkyl side chain causes decreasing E moduli (PMCA > PECA > PBCA; Table 2). Despite the examined PACA materials were in the glassy state, in which the polymer chains are frozen with restricted mobility, an increasing length of the side chains is associated with higher free volumes and less order in the packing of chains, allowing them to undergo rotation and translation upon mechanical deformation [29]. Introducing an ether group in the side chain, as shown for PMECA and PEECA, also reduced the E moduli of the polymers (Table 2). This observation for alkoxy side chains, which was more pronounced than the increasing alkyl side chain length, is associated with the lack of substitution of the chain with hydrogen atoms at oxygen ether links, again providing more free space and easier chain rotation (reducing E modulus). Future studies may investigate if potentially different molecular weights are formed during in situ polymerization of the respective PACA that might contribute to differences in *E* moduli.

### 3.3. Determination of Ultimate Properties

**Figure 3:**
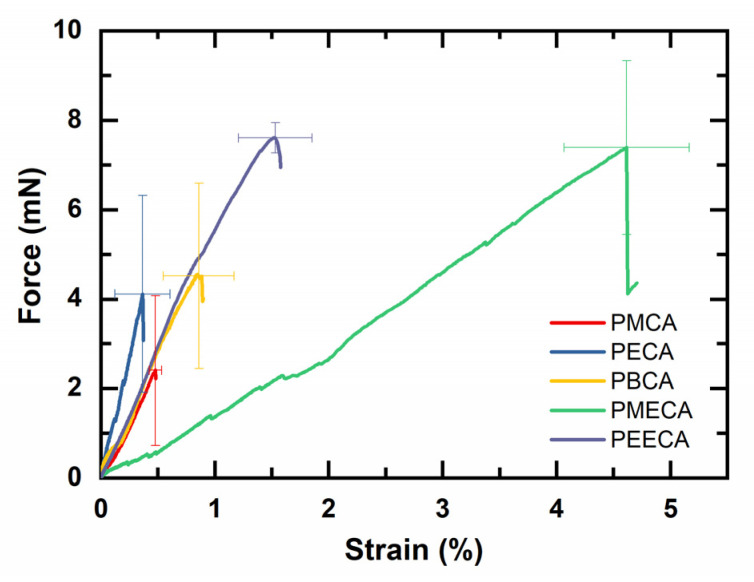
Force against strain of PACA membranes obtained by microindentation till breakage (typical experimental error of the force sensor probe is 0.5 mN). Data represented as mean ± SD (*n* = 3).

In order to determine the force at break ($F_{B}$) and the strain at break ($\varepsilon_{B}$), each PACA film was indented until rupture. The strain values were calculated from the displacement of the indenter according to Equation (5) and the force-against-strain curves are shown in Figure 3. All examined PACA films exhibited a linear deformation pattern without yield points, which confirmed their physical nature as brittle materials. The rupture of the film samples correlated with the sudden drop of the force–strain curves. 

The films made from PMCA and PECA were concluded to be the most brittle among the examined PACAs as they displayed the lowest $\varepsilon_{B}$ (≈0.4% and 0.3%, respectively). $F_{B}$ values were also low for these materials (≈2.4 mN and 4.1 mN, respectively). As PBCA has a longer and thus more flexible side chain associated with a presumably less-ordered molecular packing, the observed larger deformability of PBCA with $\varepsilon_{B}$ of 0.85% and $F_{B}$ of 4.5 mN can be justified. PACAs that contain alkoxy groups in their side chain, i.e., PMECA and PEECA, displayed an even higher $\varepsilon_{B}$ of ≈4.6 and 1.5, respectively, ($F_{B}$ ≈ 7.3 mN and ≈7.6 mN), which supported that the ether linkage in their side chains indeed enhanced the flexibility of the tested materials. 

Despite the fact that PMECA and PEECA exhibited almost similar $F_{B}$ values, PEECA had an $\varepsilon_{B}$ that was higher (≈3-fold) than PMECA. This is in agreement with a previous analysis of the adhesive strength of PEECA compared to PMECA [21]. 

During indentation, the force–strain curves of PACA films showed a minor step-drop at variable positions and intensities (compare Figure 3, e.g., for PMECA a dent at 1% strain), which might be due to small-scale crack initiation during the indentation. However, the film samples maintained their structural integrity as confirmed by continuous microscopic monitoring during the indentation experiment. The ongoing linear correlation of strain and force upon further stretching and the absence of a change in curve slope suggest that the ultimate strain was not directly correlated to the onset of microcracking.

## 4. Conclusions

In this study, a strategy to characterize the mechanical properties of free-standing films of brittle materials was proposed and evaluated, using the family of PACAs as test systems with relevance in coating applications. In agreement with previous work [21], a clear correlation of PACA side chain structure and mechanical properties both in the elastic deformation regime and as ultimate properties could be demonstrated, here using free-standing films. Multiple applications of this methodology can be suggested: for PACA materials, which can copolymerize from monomer mixture, mechanical properties may be tuned by studying structure–property relationships and selecting coatings that best fit to the applicational needs. Additionally, other materials may be deposited on the frame sample holders by casting with compatible solvents or deposition as melt, possibly after exchanging oCL with a more resistant frame material. Therefore, the here-presented methodology and data may allow to comparably investigate the mechanical properties of single or multilayer materials. Such information on the mechanical properties of films will also be helpful for modern design strategies of hybrid materials that rely on computational technics.

## Figures and Tables

**Table 1:** Chemical structures and abbreviations of used cyanoacrylate (CA) materials.

		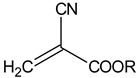		
	CA-Monomer	R	Monomer Abbreviation	PACA Abbreviation
1	Methyl CA	-CH_3_	MCA	PMCA
2	Ethyl CA	-CH_2_-CH_3_	ECA	PECA
3	Butyl CA	-CH_2_-CH_2_-CH_2_-CH_2_	BCA	PBCA
4	Methoxyethyl CA	-CH_2_-CH_2_-O-CH_2_	MECA	PMECA
5	Ethoxyethyl CA	-CH_2_-CH_2_-O-CH_2_-CH_2_	EECA	PEECA

**Table 2:** Summary of PACA film characterization. Data represented as mean ± SD for *n*= 3.

PACA	Thickness (µm) ^(a)^	$T_{g}$ (°C)	E Modulus ^(a)^ (GPa)	$F_{B}$^(a)^ (mN)	$\varepsilon_{B}$^(a)^ (%)
PMCA	6.1 ± 1.9	n.d. ^(b)^	17.1 ± 4.1	2.4 ± 1.6	0.47 ± 0.05
PECA	6.7 ± 1.5	122 ^(c)^	15.8 ± 3.0	4.1 ± 2.2	0.36 ± 0.2
PBCA	11.5 ± 2.5	99 ^(c)^	8.0 ± 1.6	4.5 ± 2.0	0.85 ± 0.3
PMECA	3.5 ± 0.5	68 ^(d)^	6.5 ± 0.5	7.3 ± 1.9	4.6 ± 0.5
PEECA	4.1 ± 1.5	62 ^(d)^	4.5 ± 0.9	7.6 ± 0.3	1.5 ± 0.3

^(a)^ Measured for films with an average thickness of 16 coating cycles. ^(b)^ Not detectable. ^(c)^ DSC heating rate 10 K·min^−1^. ^(d)^ DSC heating rate is 20 K·min^−1^. For exemplary SEM images and DSC thermograms, see Supplementary Materials, Figures S1 and S2.

## Data Availability

The data that supports the findings of this study are available within the article.

## Supplementary Materials

The following supporting information can be downloaded at: https://www.mdpi.com/article/10.3390/polym14224863/s1, Figure S1. SEM images of free-standing PACA films as isolated from square-shaped polymer frames; Figure S2. DSC thermograms of dry-state PACA samples.
