# Supplementary material for: Analyzing the Mechanical Properties of Free-Standing PACA Thin Films Using Microindentation Technique
Source: Polymers (Basel). 2022 Nov 11;14(22):4863. doi: 10.3390/polym14224863 (PMC9696112; doi:10.3390/polym14224863)
Supplement: Supplementary file 1 [file polymers-14-04863-s001.zip › polymers-2014149-supplementary.pdf]

polymers

Supplementary Materials

Analyzing the Mechanical Properties of Free-Standing PACA Thin Films Using Microindentation Technique

Osamah Altabal and Christian Wischke *,†

Institute of Active Polymers, Helmholtz-Zentrum Hereon, Kantstr. 55, 14513 Teltow, Germany
* Correspondence: christian.wischke@pharmazie.uni-halle.de; christian.wischke@hereon.de
† Current address: Institute of Pharmacy, Martin-Luther University Halle-Wittenberg, Halle, Germany.

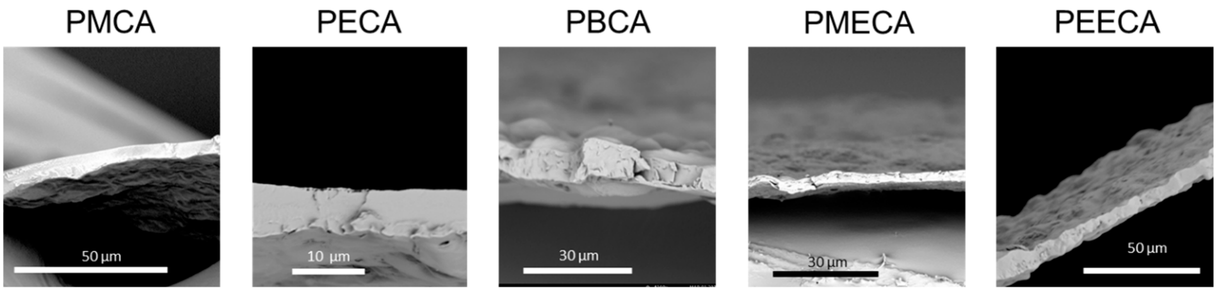

Figure S1. SEM images of free-standing PACA films as isolated from square-shaped polymer frames. For abbreviations, see main manuscript.

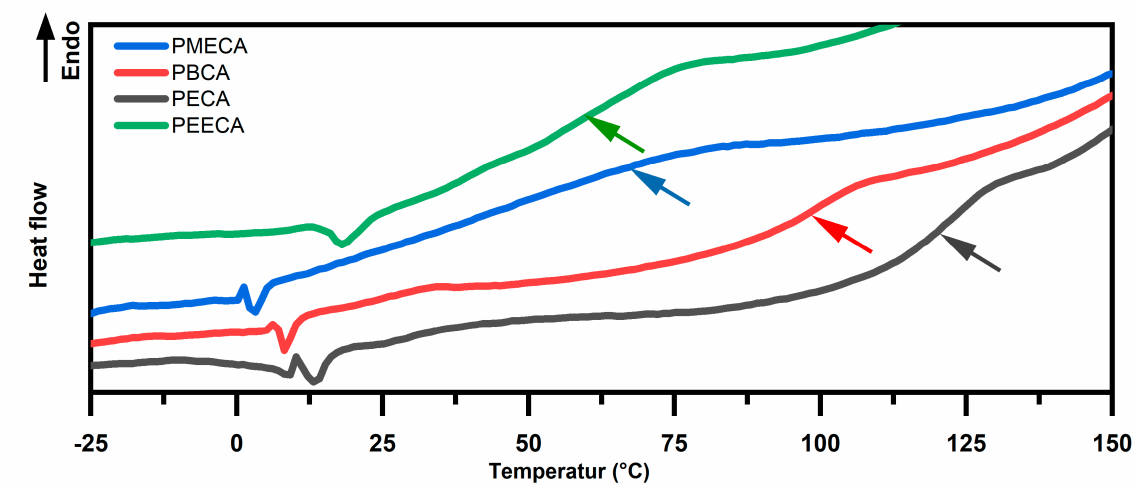

Figure S2. DSC thermograms (second heating run) of dry-state PACA samples. The signals in the range of 0-25 °C are assigned to movement of the multiple-piece samples during heating. The respective glass transition is indicated by arrows as determined by DSC software. For abbreviations, see main manuscript.
